# RNA Biology in Fungal Phytopathogens

**DOI:** 10.1371/journal.ppat.1003617

**Published:** 2013-10-17

**Authors:** Vera Göhre, Carl Haag, Michael Feldbrügge

**Affiliations:** Heinrich-Heine University Düsseldorf, Institute for Microbiology, Cluster of Excellence on Plant Sciences, Düsseldorf, Germany; The University of North Carolina at Chapel Hill, United States of America

RNA-dependent processes are essential to determine when, where, and how much of a protein is synthesized. In eukaryotes, these processes start with transcription in the nucleus and end with mRNA translation at distinct cytoplasmic sites followed by mRNA degradation [1]. In between, a number of defined steps occur such as 5′ capping, splicing, polyadenylation, nuclear export, and cytoplasmic transport. Most of the steps are tightly regulated to achieve highly precise spatiotemporal expression. RNA-binding proteins serve as key factors that are essential at each level of posttranscriptional control [1]. Small RNAs are additional factors that can inhibit translation or are able to promote degradation of mRNAs.

Due to the central importance of RNA biology in regulating protein synthesis, it is not surprising that fungal pathogens intensely rely on RNA-dependent processes to control infection. Here, we focus on RNA biology in fungal plant pathogenesis, which is best studied in the corn smut *Ustilago maydis* [2], [3].

## RNA Biology Orchestrates Fungal Infection

**Figure 1:**
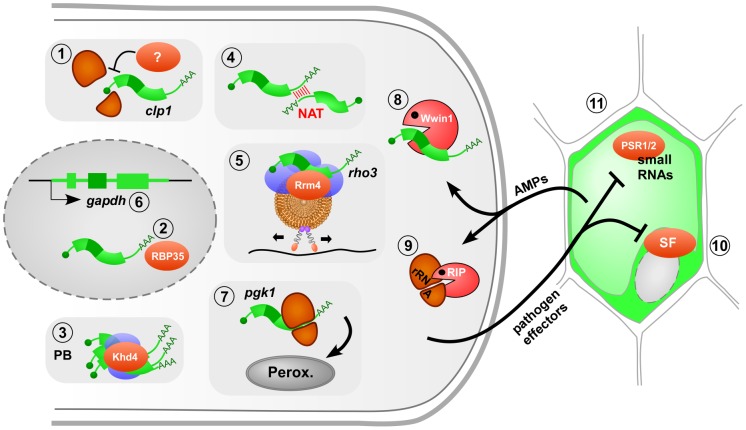
The role of fungal RNA biology during plant infection. Model of a filamentous pathogen (left) interacting with its plant host (right). RNA-dependent processes crucial for infection are numbered as follows: **1** translational regulation of *clp1* mRNA (ribosomal subunits in orange, mRNA in green); **2** 3′ end processing by the RNA-binding protein RBP35 in the nucleus (gray oval); **3** modulation of mRNA stability in processing bodies (PB, blue: accessory factors); **4** natural antisense transcripts (NAT) regulate mRNA stability; **5** endosomal mRNA transport along microtubule (black line, brown: endosome with motor proteins attached); **6** alternative splicing of *gapdh* mRNA to generate an enzyme with a peroxisomal targeting sequence; **7** translational read-through adding a peroxisomal targeting sequence; **8** antimicrobial peptide (AMP) Wheatwin1(Wwin1) with RNase activity; **9** ribosome-inactivating protein (RIP) alters rRNA; **10** pathogen effectors target host splice factors (SF) as shown for the bacterial effector HopU1 inactivating AtGRP7; **11** pathogen effectors interfere with the generation of small RNAs in the host as shown for oomycetes effectors. Further details are given in the text.

In *U. maydis*, a major pathogenicity determinant is the homeodomain transcription factor bW/bE that triggers a cascade of transcriptional responses essential for full virulence [4], [5]. Progression toward infection is regulated by several bE/bW-induced factors, including Clp1 and Cib1. Importantly, their functions are precisely regulated at the posttranscriptional level. *clp1* is a direct target gene of bW/bE, which is induced early during formation of infectious filaments. However, the Clp1 protein is only synthesized later at the specific stage of plant penetration when it acts as an antagonist of bW/bE [6]. This is indicative for a tight translational regulation of *clp1* mRNA by a currently unknown mechanism (Figure 1, #1). Cib1 constitutes a direct interaction partner of Clp1. The corresponding *cib1* gene is constitutively expressed but regulated at the level of mRNA splicing. Splicing is induced during infection, resulting in the expression of a Cib1 variant containing a functionally important Clp1 interaction domain [7]. Thus, a complex network of transcriptional and posttranscriptional controls is essential to fine-tune infection.

RNA-binding proteins are key factors in RNA biology. In the rice blast fungus *Magnaporthe grisea*, loss of the RRM (RNA recognition motif) protein RBP35 causes defects in virulence and development. Protein interaction studies revealed that RBP35 functions as a novel auxiliary protein of the polyadenylation machinery of plant pathogens (Figure 1, #2) [8]. A systematic approach in *U. maydis* deleting genes encoding various RNA-binding proteins revealed that mutants lacking Khd4 exhibit defects in morphology and pathogenicity [9]. Khd4 is a multi-KH-domain protein with homologs in other pathogens such as *Cryptococcus neoformans* and *Candida albicans*. It specifically binds the sequence AUACCC and is most likely involved in regulation of mRNA stability [10]. This is supported by the observation that Khd4 localizes to processing bodies (unpublished observation), which are known centers for mRNA degradation (Figure 1, #3). The importance of regulating mRNA stability was recently further underlined by showing that natural antisense transcripts might also be important for the regulation of mRNA stability and pathogenicity (Figure 1, #4) [11].

In addition to regulation through natural antisense transcripts, RNA interference (RNAi) is an important mechanism to control endogenous and foreign RNA in most organisms. Noteworthily, genome comparison with *Ustilago hordei* revealed that the RNAi machinery has been specifically lost in *U. maydis* during evolution, indicating that this mode of RNA regulation is clearly dispensable for a successful plant pathogen [12]. It has been rationalized that RNAi deficiency enables hosting of double-stranded RNA viruses. These can carry the genetic information to produce toxins to kill sensitive neighbors, which might account for an evolutionary advantage [13]. Taken together, posttranscriptional control at the level of splicing, polyadenylation, translation, and stability is an important regulatory tool of fungal pathogens to coordinate infection.

## mRNA Transport Is Crucial during Early Infection

In higher eukaryotes, active microtubule-dependent transport of mRNAs by molecular motors functions in spatial protein expression during developmental and neuronal processes [14]. Similarly, in *U. maydis* the ELAV (embryonic lethal abnormal vision)-type protein Rrm4 functions in long-distance mRNA transport, a process that is specifically important during formation of infectious unipolar filaments [15]–[17]. Studying the mechanism of transport revealed that shuttling of Rrm4 along microtubules is connected to membrane trafficking [17], [18]. Rrm4 colocalizes almost exclusively with motile Rab5a-positive endosomes. Similarly to endosome shuttling [19], trafficking of Rrm4 is mediated by the concerted action of the minus end–directed motor dynein Dyn1/2 and the plus end–directed kinesin Kin3 [17]. Since colocalization of Rrm4 with endosomes occurs even in the absence of Kin3, endosomal hitchhiking was proposed as mode of transport (Figure 1, #5) [17]. Importantly, Rrm4 is dispensable for endosomal shuttling, but the RNA-binding capacity of Rrm4 is needed for unipolar growth [15], [17].

Rrm4 transports specific mRNAs such as *ubi1* and *rho3*, encoding ubiquitin fused to ribosomal protein Rpl40 and the small GTPase Rho3, respectively [16]. Rho3 accumulates at retraction septa of infectious filaments and *rrm4* deletion strains are disturbed in septum formation. Hence, microtubule-dependent transport of *rho3* mRNA might act in correct septal localization of Rho3, which could promote septum insertion in infectious filaments [16].

In summary, endosomal mRNA transport along microtubules is a novel aspect of RNA biology and this posttranscriptional process is particularly important during early infection. Since the RNA-binding protein She3 functions in actin-dependent mRNA transport during infection of *C. albicans* [20], a molecular link of mRNA trafficking and infection might be widespread in fungal pathogenicity.

## RNA Biology Determines the Precise Subcellular Distribution of Proteins during Infection

Correct targeting of proteins to subcellular compartments is an essential process and defects can affect virulence. Generally proteins are directed to their subcellular location by signal sequences, whose presence can be regulated by alternative splicing of pre-mRNA. A novel example is the glycolytic enzyme glyceraldehyde-3-phosphate dehydrogenase (GAPDH) of *U. maydis*, which is usually localized in the cytoplasm. However, 10% of its mRNA is alternatively spliced, generating an enzyme with a C-terminal peroxisomal targeting sequence (PTS1 type). This results in peroxisomal localization of GAPDH, a process termed cryptic peroxisomal targeting (Figure 1, #6) [21]. Similarly, the 3-phosphoglycerate kinase (PGK) resides to a certain extent in peroxisomes. Here, ribosomal read-through of the termination codon creates an isoform with a functional C-terminal PTS1 (Figure 1, #7). The apparently “sloppy” translation accounts for the necessary fraction of peroxisomal enzyme [21]. In peroxisomes, GAPDH and PGK might function together with other NADH-dependent dehydrogenases in redox homeostasis. This regulatory process appears to be particularly important during pathogenic development, since mutants specifically lacking the peroxisomal isoforms are viable but less virulent [21]. Thus, RNA biology in form of alternative splicing and translational read-through determines the correct subcellular targeting of the encoded enzymes and this contributes to virulence.

## RNA Biology in Plant-Microbe Interactions

The importance of RNA biology in fungal infection is further underlined by the fact that posttranscriptional regulation is targeted during host defense. Plants react to infection by producing antimicrobial peptides (AMPs) that are proposed to degrade the fungal cell wall or to permeabilize the membrane. Some AMPs also enter the cell to exert their function [22]. An important example for the latter is Wheatwin1, a pathogenesis-related protein (PR4) with antifungal activity [23]. Wheatwin1 is targeted to the cytoplasm of intruding fungi where it functions as RNase and inhibits pathogenic growth by hydrolyzing fungal RNA (Figure 1, #8) [23]. Furthermore, plants express ribosome-inactivating proteins with antifungal activity. These attack ribosomes by exhibiting N-glycosidase activity, removing a single adenine from the rRNA, and thereby translation is inhibited (Figure 1, #9) [24]. Thus, plants interfere with fungal RNA biology for defense.

Intriguingly, the opposite is true as well: pathogens interfere with posttranscriptional control of the host to promote infection. Although this has so far only been shown for bacterial and oomycete phytopathogens [25], we predict that similar mechanisms will be discovered for fungal phytopathogens. For example, it has been known for many years that *U. maydis* secretes ribonucleases that might function as virulence factors in degrading host RNAs [26]. A prominent case for bacterial effectors is HopU1 from *Pseudomonas syringae* that is essential for virulence on *Arabidopsis thaliana* [27]. HopU1 functions as mono-ADP-ribosyltransferase and modifies the RNA-binding domain of AtGRP7, a splice factor that regulates expression of the immune receptor FLS2 (Figure 1, #10). Thus, bacterial effectors appear to inactivate host RNA-binding proteins by posttranslational modification to overcome plant defense.

Moreover, microbial effectors interfere with plant microRNA activity. miR393, for instance, targets the F-box auxin receptor TIR1 and thereby represses auxin signaling, a process involved in plant immunity [28]. The bacterial effector AvrPto interferes with microRNA function in the plant host both at the transcriptional and posttranscriptional level [29]. This seems to be a broad concept also present in oomycete pathogens. The effector PSR1 inhibits DICER-dependent functions and thereby it suppresses biogenesis of a broad range of mi- and siRNAs involved in regulating plant immunity. The effector PSR2 specifically targets a subset of trans-acting siRNAs, resulting in altered expression of host resistance proteins (Figure 1, #11) [30]. In summary, plant defense targets fungal RNA biology and *vice versa* pathogen effectors suppress host RNA silencing during plant protection.

## Conclusions and Future Directions

The different levels of RNA biology such as alternative splicing, 3′ end processing, endosomal mRNA transport, and translational regulation are essential to orchestrate plant infection of fungal pathogens. RNA-binding proteins are key components in executing these functions. After improving transcriptome-wide approaches such as iCLIP [31], we are now able to investigate the complete RNA-binding landscape of RNA-binding proteins involved in fungal infection [32]. Moreover, it has become apparent that RNA biology is also particularly important during plant/microbe communication. Mutually exchanged effectors act on RNA components of the defence/virulence pathway of the partner. Thus, RNA biology emerges as an important research field with the potential to identify new Achilles' heels of fungal pathogens. Taking plants as an inspiring model, drugs interfering with RNA-dependent processes of pathogens should be considered for host protection in the future.
